# Influence of extending expansive open-door laminoplasty to C1 and C2 on cervical sagittal parameters

**DOI:** 10.1186/s12891-020-3083-1

**Published:** 2020-02-05

**Authors:** Wen-xuan Wang, Yi-bo Zhao, Xiang-dong Lu, Xiao-feng Zhao, Yuan-zhang Jin, Xian-wei Chen, Yan-xin Fan, Xiao-nan Wang, Run-tian Zhou, Bin Zhao

**Affiliations:** Department of Orthopaedics, The Second Hospital of Shanxi Medical University, NO.382, Wuyi Road, Taiyuan, 030001 Shanxi China

**Keywords:** Upper cervical spine, Muscular-ligament complex, Expansive open-door laminoplasty, Cervical sagittal parameters

## Abstract

**Background:**

For patients with spinal canal stenosis in the upper cervical spine who undergo C3–7 laminoplasty alone, it remains impossible to achieve full decompression due to its limited range. This study explores the extension of expansive open-door laminoplasty (EODL) to C1 and C2 for the treatment of cervical spinal stenosis of the upper cervical spine and its effects on cervical sagittal parameters.

**Methods:**

A retrospective analysis of 33 patients presenting with symptoms of cervical spondylosis myelopathy (CSM) and ossification in the posterior longitudinal ligament (OPLL) of the upper cervical spine from February 2013 to December 2015 was performed. Furthermore, the changes in the C0–2 Cobb angle, C1–2 Cobb angle, C2–7 Cobb angle, C2–7 SVA, and T1-Slope in lateral X-rays of the cervical spine were measured before, immediately after, and 1 year after the operation. JOA and NDI scores were used to evaluate spinal cord function.

**Results:**

The C0–2 and C1–2 Cobb angles did not significantly increase (*P* = 0.190 and *P* = 0.081), but the C2–7 Cobb angle (*P* = 0.001), C2–7 SVA (*P* < 0.001), and T1-Slope (*P* < 0.001) significantly increased from preoperative to 1 year postoperative. In addition, C2–7 SVA was significantly correlated with the T1-Slope (Pearson = 0.376, *P* < 0.001) and C0–2 Cobb angle (Pearson = 0.287, *P* = 0.004), and the C2–7 SVA was negatively correlated with the C2–7 Cobb angle (Pearson = − 0.295, *P* < 0.001). The average preoperative and postoperative JOA scores were 8.3 ± 1.6 and 14.6 ± 1.4 points, respectively, indicating in a postoperative neurological improvement rate of approximately 91.6%. The average preoperative and final follow-up NDI scores were 12.62 ± 2.34 and 7.61 ± 1.23.

**Conclusions:**

The sagittal parameters of patients who underwent EODL extended to C1 and C2 included loss of cervical curvature, increased cervical anteversion and compensatory posterior extension of the upper cervical spine to maintain visual balance in the field of vision. However, the changes in cervical spine parameters were far less substantial than the alarm thresholds reported in previous studies. We believe that EODL extended to C1 and C2 for the treatment of patients with spinal canal stenosis in the upper cervical spine is a feasible and safe procedure with excellent outcomes.

## Background

Posterior surgery, which includes laminoplasty and laminectomy, is recognized as a safe and effective indirect decompression approach. Most spinal surgeons accept cervical EODL because of the simplicity of the operation, the immediate postoperative stability of the cervical vertebra and the reliability of its long-term clinical efficacy [1–3]. However, in our clinical practice, some patients with spinal canal stenosis in the upper cervical spine who underwent only C3–7 laminoplasty did not achieve full decompression due to its limited range. In addition, in some cases, new-onset compression or folding angle occurred during the postoperative shift of the spinal cord. In such situations, patients may experience poor improvement of symptoms or repeated aggravation after temporary relief. Accordingly, in the present study, we explored the effects of extending EODL to C1 and C2 for the treatment of cervical spinal stenosis of the upper cervical spine. EODL may damage the posterior cervical muscle-ligament complex during lamina exposure [4, 5], and this may lead to the loss of cervical stability, postoperative axial symptoms and even kyphosis deformity, which ultimately affects patient quality of life.

In this study, we retrospectively analysed patients who underwent EODL extended to C1 and C2 at our hospital and measured sagittal parameters of the cervical spine before, immediately after and 1 year after the operation during follow-up. The purpose of this study was to investigate changes in sagittal parameters of the cervical spine and the effect of EODL extended to C1 and C2 on cervical stability.

## Methods

### General data

A retrospective analysis was performed with a focus on patients with cervical myelopathy caused by ossification of the upper cervical spine who underwent posterior cervical EODL from February 2013 to December 2015 at our hospital. This study was approved by the IRB of the authors’ affiliated institutions. The inclusion criteria were as follows: (1) patients with spinal canal stenosis in the upper cervical spine; (2) patients with cervical myelopathy due to cervical ossification confirmed by imaging data (X-ray, CT and MRI); and (3) patients who had been followed up for at least 12 months after the operation. Exclusion criteria included (1) patients with instability of the upper cervical spine; (2) patients with congenital deformity of the upper cervical spine; (3) patients with trauma, infection or a tumour; and (4) patients with a previous history of cervical spine surgery.

A total of 33 patients, including 22 males and 11 females, were enrolled. Their ages ranged from 48 to 76 years old [average of 60.1 ± 7.0 years old] and the disease course ranged from 6 to 84 months [average of 28 ± 15 months]. All patients were successfully followed up.

### Surgical approach

**Fig. 1:**
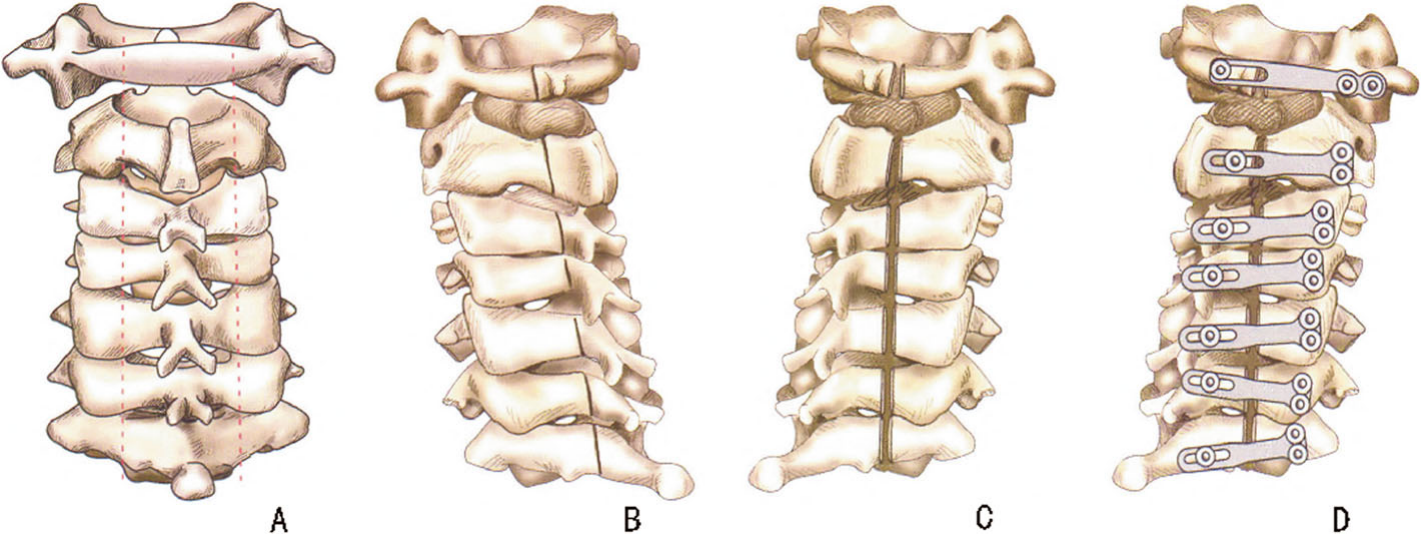
**a** Exposure of the C1-C7 posterior arch and lamina. The spinous arch is removed, and grooves are made at the junction of the lamina and lateral mass. **b** The door-oriented cortical bone of the inner plate was retained. When performing C1 laminoplasty, a slot was made between the posterior arch and lateral mass. **c** Excision of the ligamentum flavum, adhesive dura mater and vascular bridge was performed to achieve an appropriate opening angle and adequate decompression of the spinal cord. **d** Titanium miniplates are placed to secure the elevated laminae. An I-type mini-titanium plate was used in C1 laminoplasty, and a T-type mini-titanium plate was applied in C2-C7 laminoplasty

The patients were instructed to keep the prone position with their head fixed on the Mayfield head rack. The standard posterior median approach was used to expose the C1-C7 posterior arch and lamina and extend exposure to the facet joint on the lateral side. The supraspinal ligaments, spinous processes, and interspinal ligaments were removed; moreover, grooves were made at the junction of the lamina and lateral mass with a 2 mm rongeur to remove the cortex and cancellous bone. On the incision side, the cortex of the inner lamina was removed with a 1 mm laminectomy rongeur. The door-oriented cortical bone of the inner plate was retained until the posterior structure could be easily rotated to the dorsal side. Excision of the ligamentum flavum, adhesive dura mater and vascular bridge was performed to achieve an appropriate opening angle and adequate decompression of the spinal cord. Next, the posterior atlanto-occipital membrane was carefully resected at the occipitocervical level. After adequate decompression, the pulsation of the dura mater should suggest a satisfactory decompression effect. Subsequently, the mini-titanium plate for posterior cervical laminoplasty was placed between the lamina and the open side of the lateral mass. Neuromonitoring was utilized in all procedures. All patients were operated on by the same group of surgeons. The operation method is shown in Fig. 1a-d.

### Imaging evaluation

**Fig. 2:**
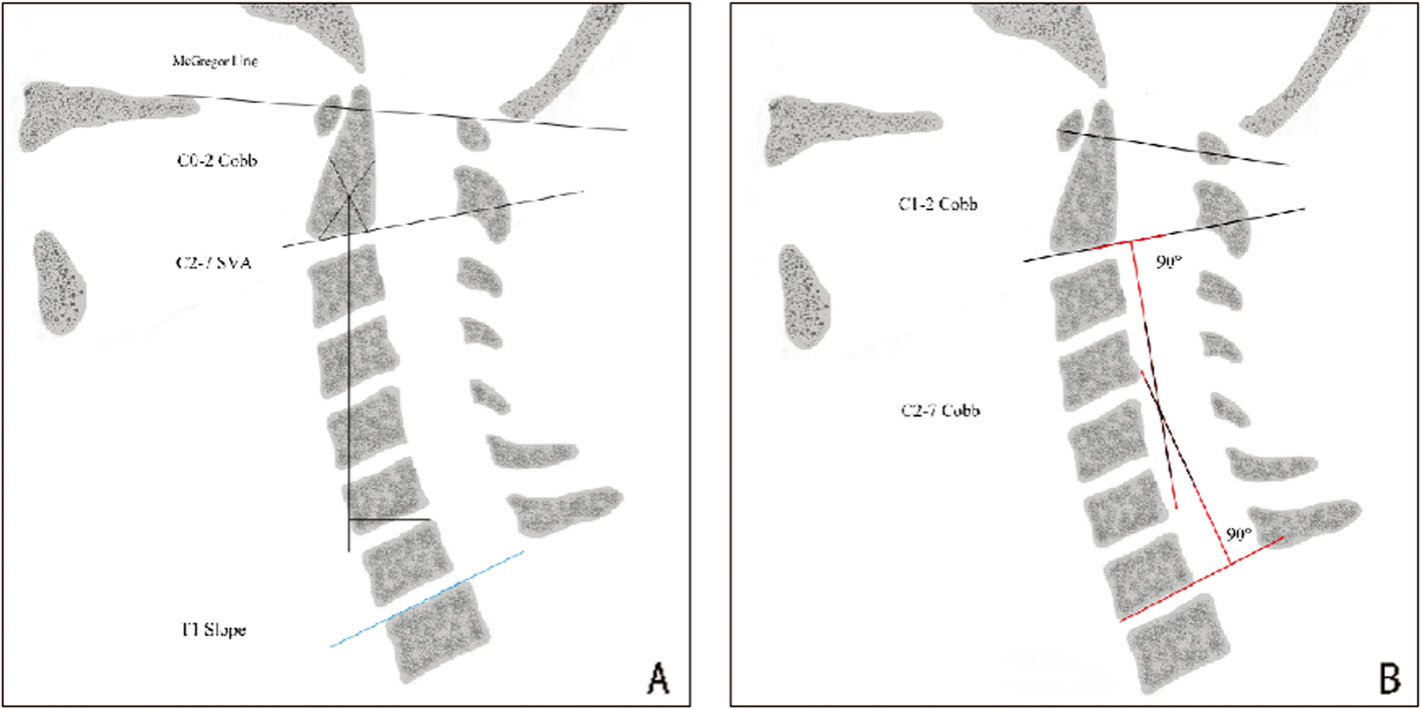
**a** C0–2 Cobb angle: the intersection angle between the McGregor line and the parallel line of the C2 lower endplate, which is used to evaluate the curvature of the upper cervical spine. C2–7 SVA: horizontal distance from the vertical line of the C2 vertebral geometric centre to the posterior edge of the C7 vertebral upper endplate. T1-Slope: the intersection angle between the tangent line and the horizontal line of the upper endplate of the T1 vertebral body. **b** C1–2 Cobb angle: the intersection angle between the midpoint line of the anterior and posterior arch of the atlas and the line parallel to the lower endplate of C2. C2–7 Cobb angle: the intersection angle between the line perpendicular to the line parallel to the C2 lower endplate and the line perpendicular to the line parallel to the C7 lower endplate; this measurement is applicable for assessing lower cervical curvature, including kyphosis (C2–7 Cobb angle < 0°), a physiological curvature that becomes straight (0°~ 10°) and lordosis (C2–7 Cobb angle > 10°)

The imaging parameters of the lateral X-ray of the cervical spine were measured preoperatively, postoperatively and at the last follow-up. The evaluated imaging parameters included ① the occipito-cervical angle (C0–2 Cobb angle), which is the intersection angle between the McGregor line and the line parallel to the C2 lower endplate and is used to evaluate the curvature of the upper cervical spine; ② the C2–7 Cobb angle, which is the intersection angle between the line perpendicular to the line parallel to the C2 lower endplate and the line perpendicular to the line parallel to the C7 lower endplate; ③ the C1–2 Cobb angle, which is the intersection angle between the midpoint line of the anterior and posterior arch of the atlas and the line parallel to the lower endplate of C2; ④ the C2–7 sagittal vertical alignment (C2–7 SVA), which is the horizontal distance from the line vertical to the C2 vertebral geometric centre to the posterior edge of the C7 vertebral upper endplate; and ⑤ the T1-Slope, which is the intersection angle between the tangent line and the upper plate of the T1 vertebral body. All imaging parameters were measured by three spine surgeons using measurement software (Surgimap Spine Nemaris Inc.). The measurement methods are shown in Fig. 2a-b.

### Statistical analysis

SPSS 21.0 was used for statistical analysis. Experimental data are expressed as the mean ± standard deviation ($\overline{x}\pm s$) and were statistically analysed by completely randomized two independent samples t-tests. Pearson’s correlation analysis was performed to evaluate the correlation between the two variables. The observed difference was statistically significant at *P* < 0.05.

## Results

### Sagittal parameters of the upper cervical spine

In this study, 10 patients underwent C1–7 EODL, and 23 underwent C2–7 EODL (All patients parameter data were in Additional file 1). Briefly, in patients who underwent C1 and C2 EODL, the C0–2 Cobb angle increased from 19.58 ± 6.06° preoperatively to 21.38 ± 4.95° at the last follow-up; however, there was no significant difference between the two groups (*P* = 0.190). In addition, there was no significant difference in the C1–2 Cobb angle between preoperative and final follow-up measurements [(22.06 ± 6.00)° vs. (24.50 ± 5.09)°; *P* = 0.081] (Tables 1 and 2).

**Table 1:** Comparison of the cervical spine sagittal parameters between pre-operation and post-operation group

Parameters	Preoperative	Postoperative	t value	*P* value
C0–2	19.58 ± 6.06	20.96 ± 5.65	− 0.954	0.344
C1–2	22.06 ± 6.00	23.29 ± 4.42	−0.946	0.348
C2–7	22.05 ± 5.07	20.35 ± 7.40	1.091	0.279
C2–7SVA	13.05 ± 3.79	21.87 ± 5.96*	−7.167	<0.001
T1 Slope	16.53 ± 6.72	22.38 ± 5.79*	−3.790	<0.001

*P < 0.05

**Table 2:** Comparison of the cervical spine sagittal parameters between pre-operation and final follow-up group

Parameters	Preoperative	Final follow-up	t value	*P* value
C0–2	19.58 ± 6.06	21.38 ± 4.95	−1.325	0.190
C1–2	22.06 ± 6.00	24.50 ± 5.09	−1.755	0.081
C2–7	22.05 ± 5.07	16.55 ± 7.11*	3.618	0.001
C2–7SVA	13.05 ± 3.79	22.86 ± 6.49*	−7.494	<0.001
T1 Slope	16.53 ± 6.72	24.14 ± 4.93*	−5.248	<0.001

*P < 0.05

### Sagittal parameters of the lower cervical spine

C2–7 SVA and T1-Slop were significantly higher immediately after the operation and at the last follow-up than before the operation. The average C2–7 SVA was 21.87 ± 5.96 mm postoperative (*P* < 0.001) and 22.86 ± 6.49 mm at the last follow-up (P < 0.001). The average T1-Slop was (22.38 ± 5.79)° immediately after the operation (*P* = 0.011), which was approximately 6° higher than the measurements obtained before the operation (16.53 ± 6.72)°, and had increased to (24.14 ± 4.93)° at the last follow-up (*P* = 0.001). Additionally, the average C2–7 Cobb angle was (22.05 ± 5.07)° before the operation and decreased to (20.35 ± 7.40)° after the operation (*P* = 0.279); this difference was not significant. However, 1 year after the operation, the C2–7 Cobb angle had decreased to an average of 16.55 ± 7.11° (*P* = 0.001).

### Improvement in neurological function

The JOA score increased from 8.3 ± 1.6 preoperatively to 14.6 ± 1.4 postoperatively, resulting in a JOA improvement rate of 91.6%. Neurological function significantly improved.

The NDI scores were 12.62 ± 2.34 preoperatively and 7.61 ± 1.23 at the final follow-up, and the average score was 4.3 points lower than preoperative (*P* = 0.002).

### Correlation analysis

The C2–C7 SVA was positively correlated with the T1 slope (Pearson = 0.376, P<0.001) and negatively correlated with the C2–7 Cobb angle (Pearson = − 0.295, P<0.001). Additionally, there was a positive correlation between the C0–2 Cobb angle and the C2–7 SVA (Pearson’s correlation = 0.287, *P* = 0.004) (Table 3).

**Table 3:** Pearson correlation of the parameters

	C1–2	C2–7	C2–7 SVA	T1 Slope
C0–2	−0.156	−0.133	0.287^a^	0.182
C1–2		−0.152	0.168	−0.107
C2–7			−0.295^a^	−0.17
C2–7 SVA				0.376^a^

^a^ Significant correlation at the 0.01 level (2 tailed)

## Discussion

### Impact of EODL extended to C1 and C2 on sagittal parameters of the upper cervical spine

Over the past few years, sagittal parameters of the cervical spine have received increasing attention*,* especially with regard for evaluating surgical efficacy and quality of life. Among these parameters, sagittal imbalance is considered the leading cause of poor prognosis and functional loss in patients. Among sagittal parameters, the C0–2 Cobb angle is usually used to evaluate the curvature of the upper cervical spine. Ota et al. [6] found that changes in the C0–2 Cobb angle have a strong linear correlation with changes in the narrowest oropharyngeal airway space (nPAS). In addition, some studies [7–9] have reported that postoperative dyspnoea and/or dysphagia are closely related to a decrease in the C0–2 Cobb angle.

The C1–2 Cobb angle is regarded as a pivotal factor for determining the curvature of the lower cervical spine since excessive C1–2 protrusion can potentially give rise to sagittal kyphosis of the lower cervical spine after surgery [10]. During the follow-up of 119 patients with atlantoaxial instability caused by the separation of the odontoid process, Yudoyono and his team [11] discovered a linear correlation between changes in C1–2 lordosis and C2–7 kyphosis both before and after the operation. Furthermore, Guo and colleagues [12] found that the C1-C2 Cobb angle is negatively correlated with the C2-C7 Cobb angle and that the former index is the crucial factor that affects the curvature of the lower cervical spine in operations involving atlantoaxial joint fixation. He also believed that the optimum C1–2 Cobb angle should be between 25° and 30°. Similarly, Wang et al. [13] confirmed that when the sequence of the upper cervical spine changes, the lower cervical spine will compensate for excessive lordosis or kyphosis to maintain body balance and horizontal vision. We believe an increase in the C1–2 Cobb angle may further accelerate the degeneration of the anterior longitudinal ligament, the intervertebral disc and facet joints, and these changes may, in turn, lead to the sagittal imbalance of the cervical spine.

In this study, none of the patients developed postoperative dyspnoea and/or dysphagia. We observed an increase in the C0–2 Cobb and C1–2 Cobb angles, and there was a positive correlation between the C0–2 Cobb angle and C2–7 SVA (Pearson = 0.287, *P* = 0.004). We propose that this may be due to a compensatory mechanism by which the body maintains visual or body balance. However, there were slight, non-significant increases in the C0–2 Cobb (*P* = 0.190) and C1–2 Cobb (*P* = 0.081) angles, indicating that extending EODL to C1 and C2 did not damage the stability of the upper cervical spine.

### The impact of extending EODL to C1 and C2 on sagittal parameters of the lower cervical spine

**Fig. 3:**
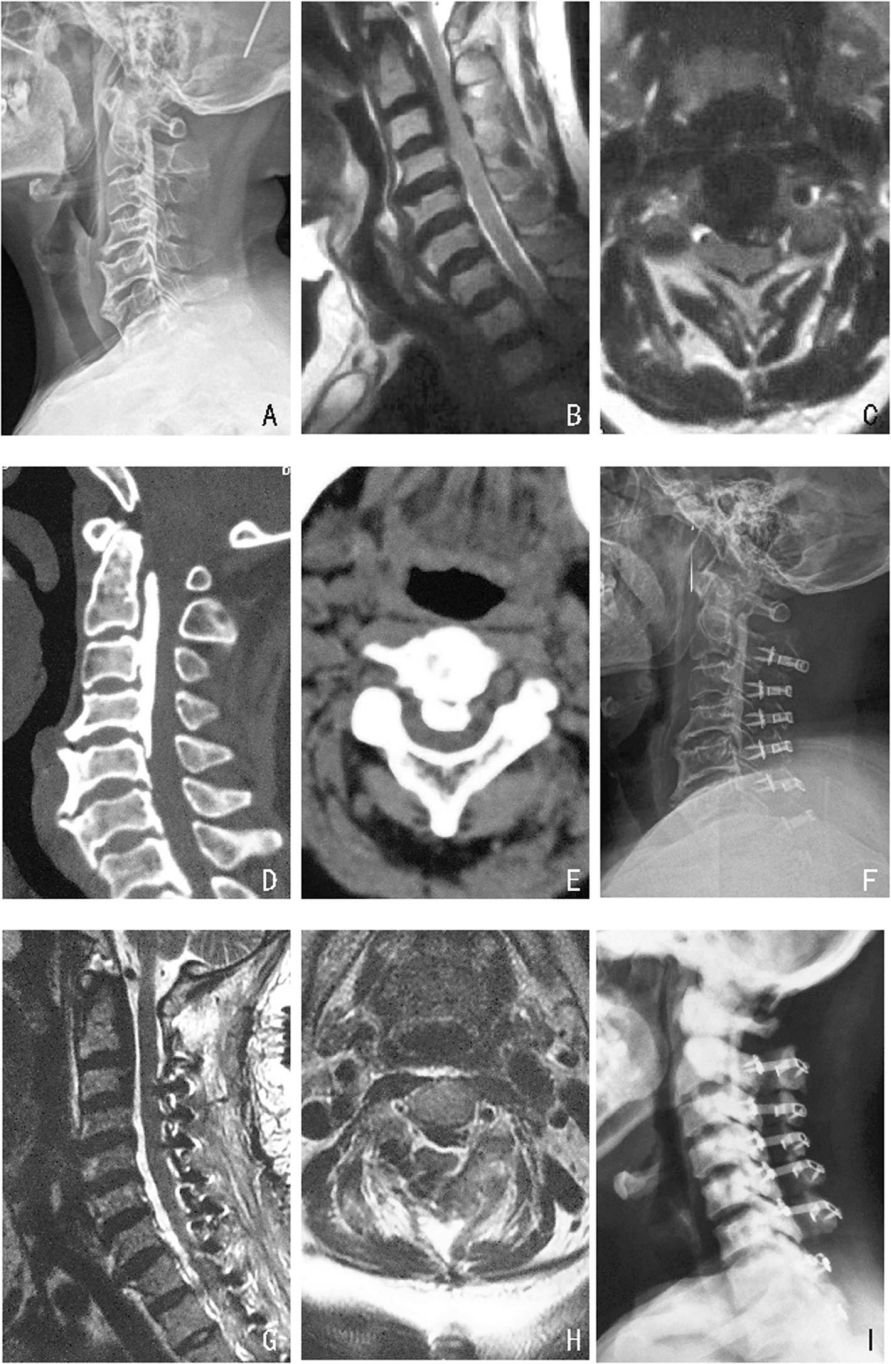
**a**, **f**, **i** A comparison of the changes in cervical alignment observed between measurements among preoperative, postoperative and at 1 year after the operation in radiographs pf a 54-year-old female patient who underwent EODL Extended to C2. **b**-**e** Preoperative MRI and CT. **g**-**h** Postoperative MRI

The C2–7 Cobb angle, C2–7 SVA, and T1-Slope are important parameters that are used to measure the sagittal balance of the cervical spine. In this study, the C2–7 Cobb angle significantly decreased, while the C2–7 SVA and T1-Slope obviously increased postoperatively, indicating a trend toward forward inclination of the cervical spine. Ames et al. [14] found that all spine segments interact with each other. Consequently, a series of changes in the sagittal sequence of the spine can be regarded as a compensatory mechanism to adjust the sagittal balance of the spine. Previous studies have recommended C2–7 SVA as one of the most important parameters for predicting the outcome of surgery. Tang and his team [15] reported that C2–7 SVA was positively correlated with the NDI index and negatively correlated with SF-36 scores in 113 patients who underwent open-door surgery of the cervical spine. The researchers suggested that the increase in SVA observed in patients with an abnormal sagittal sequence in the cervical spine is one of the causes of their poor scores for health-related quality of life. Tang assumed that when the C2–7 SVA exceeds a threshold of 40 mm, quality of life will markedly decline. Furthermore, Mohanty et al. [16] suggested that the cervical spine is in the anteversion position when C2–7 SVA is increase and that a decrease in spinal canal volume and the available space in the spinal cord on MRI might be the cause of poor outcomes after the operation. In our study, although the C2–7 SVA increased to an average of 22.86 ± 6.49 mm after cervical surgery, this was much lower than the threshold of 40 mm proposed by Tang et al. Although an anteversion trend was observed after cervical surgery, no decrease in clinical efficacy was found during follow-up (Fig. 3).

As a predictor of the sagittal balance of the cervical spine, the T1-Slope is strongly correlated with cervical curvature. Knott [17] suggested that there might be a positive and negative sagittal imbalance when the T1-Slope is greater than 25° or lower than 13° on a lateral X-ray of the cervical spine, and the sagittal sequence should be comprehensively evaluated in these patients. Our data indicate that there is a significant positive correlation between the T1-Slope and C2–7 SVA, consistent with the report by Knott and his team. In addition, our experimental results showed that T1-Slope can be used as an important indicator for evaluating sagittal balance, predicting clinical effects after surgery and directing orthopaedic programs.

### Causes of imbalance after cervical surgery

Some studies have reported on the treatment of upper cervical spinal stenosis. Matsuzaki et al. [18] used dome-like expansive laminoplasty for the second cervical vertebra. Kim [19] introduced a new surgical technique for C1–C2 fusion combined with C1 double-door laminoplasty to treat patients with C1–C2 instability, canal stenosis, and cervical spondylotic myelopathy. Zhang et al. [20] reported on the results of tension-band laminoplasty (TBL) performed with/without simultaneous C1 laminectomy and found that performing laminoplasty with simultaneous C1 laminectomy results in a greater posterior spinal cord shift than was observed for laminoplasty alone. In addition, Kong reported that 17 patients who underwent C3–7 or C2–7 open-door laminoplasty without alleviation or aggravation of spinal cord injury symptoms underwent reoperation with decompression upward to the C1 level. Due to the extended range of decompression, the spinal cord was fully decompressed, achieving satisfactory clinical effects. However, loss of cervical curvature occurred in 17 patients, 2 of whom had cervical lordosis that straightened and 1 straight case that developed kyphosis [21].

The spine is the central axis that supports the skull and trunk, and its stability and mobility are its major functional characteristics. According to the three-column theory of the cervical spine proposed by Louis [22], the anterior column, which is composed of the centrum and intervertebral disc, accounts for 36% of the load distribution generated by head weight, while the two posterior columns, which are composed of facet joints, account for a combined 64% of the load. Nolan [23] and Sherkl [24] stated that the cervical ligament complex, which is composed of the spinous process, interspinous ligament and supraspinal ligament and attached muscles, is the predominant factor that maintains the static stability of the cervical spine.

**Fig. 4:**
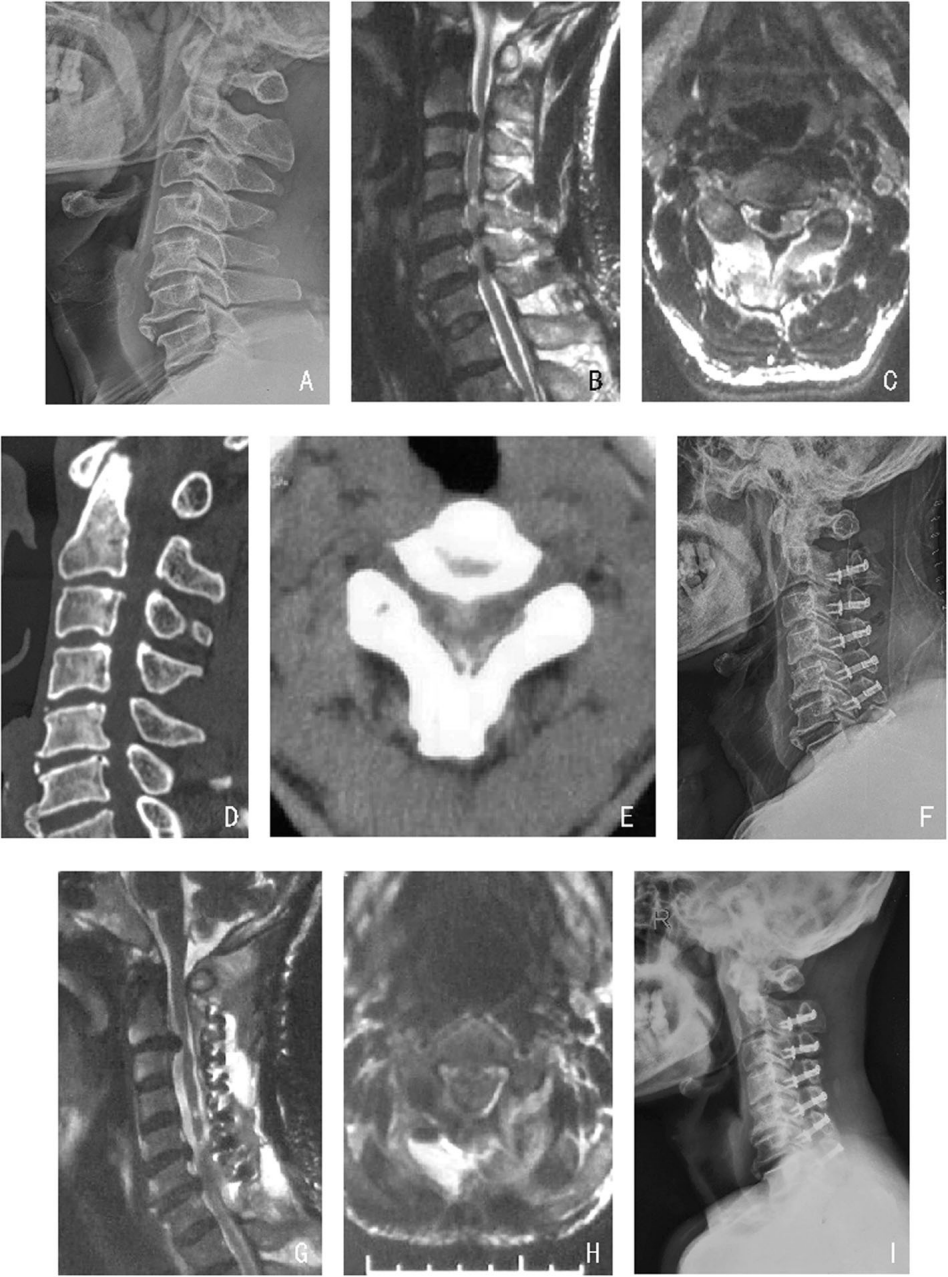
**a**, **f**, **i** A comparison of the changes in cervical alignment among measurements obtained preoperative, postoperative and 1 year after operation in a 52-year-old male patient who received EODL Extended to C2. **b**-**e** Preoperative MRI and CT. **g**-**h** Postoperative MRI. The C2–7 Cobb angle decreased after the operation, and the C2–7 SVA increased by 6.2 mm

**Fig. 5:**
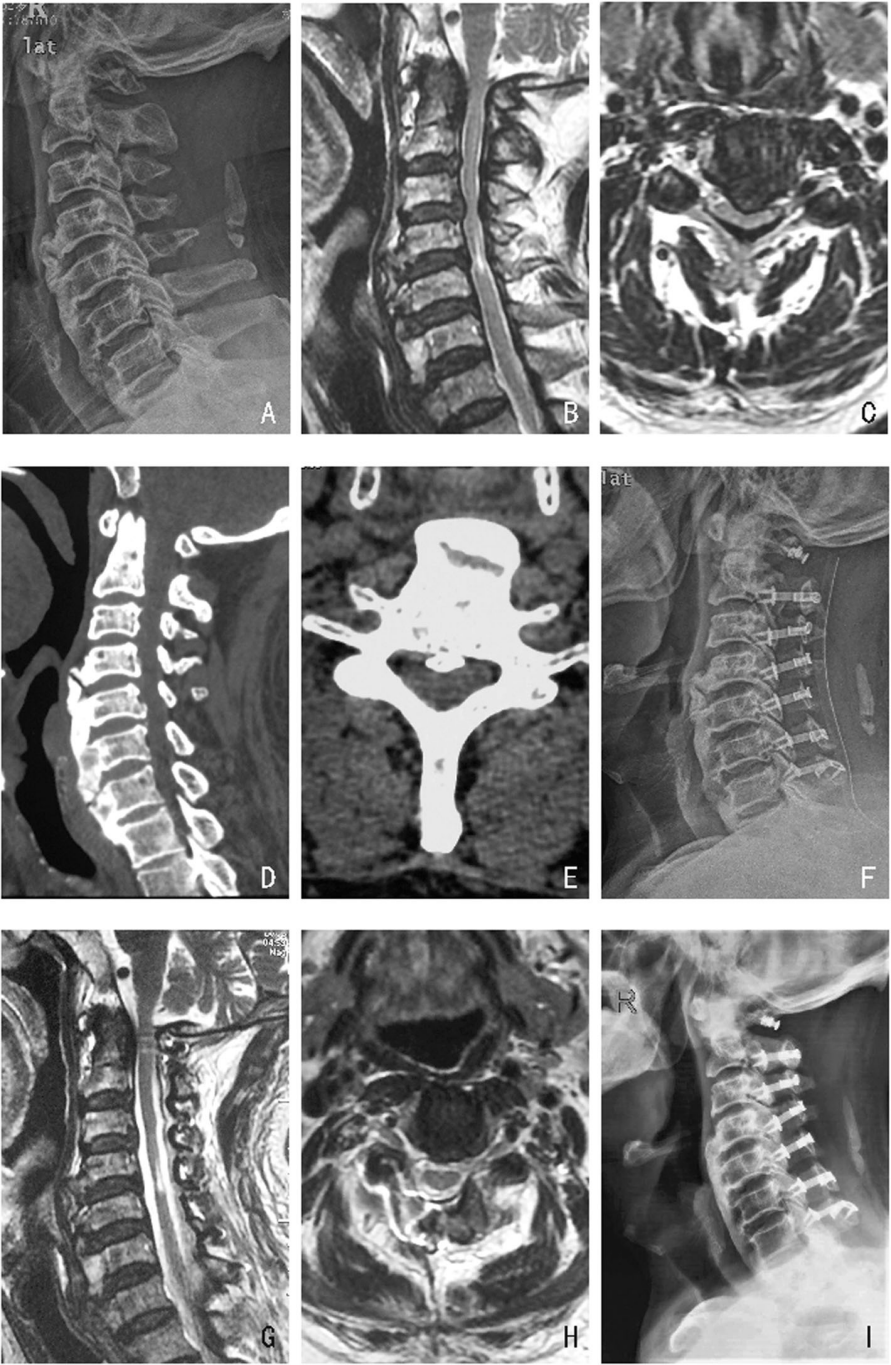
**a**, **f**, **i** A comparison of the changes in cervical alignment among measurements obtained preoperative, postoperative and 1 year after surgery in radiographs obtained in a 62-year-old male patient who underwent EODL extended to C1. **b**-**e** Preoperative MRI and CT. **g**-**h** Postoperative MRI

As the EODL requires the dissection of the bilateral paravertebral muscles and the cutting of the extensive posterior ligament structure, the resulting invasion will accelerate muscle fatigue, which could potentially bring about sagittal imbalance of the cervical spine, instability of the cervical vertebra and a decline in quality of life [25]. Accordingly, in this experiment, the suture method was modified by suturing the fascia layer only on the back to ensure the muscle’s central axis movement and retain the facet joint on the lateral side as far as possible. On the second day after the operation, the patients could stand and walk by wearing a collar, and they were encouraged to undergo progressive tolerance training during flexion, extension, rotation and lateral bending. It was recommended that the neck collar be worn within 2 weeks after the operation. Moreover, a self-designed titanium mini-plate was applied to reconstruct the lamina structure and achieve “rigid fixation,” increase cervical stability and prevent the “re-closing” of the lamina structure. During the follow-up, patients in our study achieved good clinical outcomes (Figs. 4 and 5).

Vertebral artery injury is a severe complication of upper cervical spine surgery. Although its incidence is low, affected patients may be at risk due to significant blood loss. Therefore, to avoid damaging the blood vessels and nerves in the vertebral artery sulcus, surgeons should allow an appropriate distance and be cautious when exposing or resecting the posterior arch of the atlas [26]. The author’s experience in imaging analysis and surgical summary of the posterior arch of the atlas suggests the following. (1) The posterior arch of the atlas has different resections and exposure ranges and should be treated differently. Because the posterior arch is fan-shaped and has a certain degree of curvature, the median medial distance should be used as the reference value when removing the posterior arch and should not exceed 12 mm. If it exceeds 12 mm, care should be taken to avoid damaging the blood vessels and nerves in the vertebral artery groove. (2) The posterior arch exposure should be controlled at 12 mm, and the posterior arch resection should be limited to 8 mm. (3) The posterior arch of the atlas should be stripped under the periosteum, with the posterior and lower part regarded as the safe area. After the vertebral artery is exposed, the posterior arch can be removed directly. (4) There are large individual differences in the safe area of the posterior arch of the atlas. Adults are larger than children, and males are larger than females. Therefore, posterior arch resection should be accurately individualized to each patient.

## Conclusion

The sagittal parameters of patients who underwent EODL extended to C1 and C2 changed, including a loss of cervical curvature, increased cervical anteversion and compensatory posterior extension of the upper cervical spine to maintain visual balance in the field of vision. However, the changes in cervical spine parameters were far less marked than the alarm thresholds reported in previous studies. We believe that EODL extended to C1 and C2 for the treatment of patients with spinal canal stenosis in the upper cervical spine is a feasible and safe procedure that provides excellent outcomes. Future studies should focus on the exploration of an effective approach to preserve the posterior cervical muscle-ligament complex to the greatest extent and to maintain the sagittal balance of the cervical spine.

## Supplementary information

**Additional file 1.** The parameter data for all the patients.

## Data Availability

All data generated or analyzed during this study are included in this published article and its additional files.

## Abbreviations

CSM: Cervical spondylosis myelopathy
CT: Computed tomography
EODL: Expansive open-door laminoplasty
JOA: Japanese Orthopaedic Association Scores
MRI: Magnetic resonance imaging
NDI: Neck disability index
OPLL: Ossification in the posterior longitudinal ligament
TBL: Tension-band laminoplasty
